# Supplementary material for: The Use of Galactomannan Antigen Assays for the Diagnosis of Invasive Pulmonary Aspergillosis in the Hematological Patient: A Systematic Review and Meta-Analysis
Source: J Fungi (Basel). 2023 Jun 15;9(6):674. doi: 10.3390/jof9060674 (PMC10305130; doi:10.3390/jof9060674)
Supplement: Supplementary file 1 [file jof-09-00674-s001.zip › jof-2374204-supplementary.pdf]

Supplementary File S1 – Search strategy

	**Number before deduplicate**	**Number after deduplicate**
**Pubmed**	281	281
**Embase**<br>**Of which conf. abstr.**	1145<br>360	881<br>354
**Cochrane**	32	15
**Total**<br>**Without conf. abstr.**	1458	1177<br>824

Deduplicate

	Duplicates	Pubmed	Embase	Cochrane
		281	1145	32
A	44	281	1106	29
B	126	281	981	26
C	42	281	940	25
D	9	281	931	25
E	2	281	929	25
F	51	281	888	17
G	7	281	882	16
By hand	2	281	881	15
**Total**	**279**			

PUBMED - 13-04-2021

Search number	Query	Results
4	#1 AND #2 AND #3	281
3	"galactomannan" [Supplementary Concept] OR Galactomannan[tiab]	2,442
2	"Hematologic Diseases"[Mesh] OR "Hematologic Neoplasms"[Mesh] OR "Leukemia"[Mesh] OR "Lymphoma"[Mesh] OR "Multiple Myeloma"[Mesh] OR lymphoma*[tiab] OR myeloma*[tiab] OR "myeloidos*"[tiab] OR "myelopathy*"[tiab] OR hodgkin*[tiab] OR nonhodgkin*[tiab] OR leukemi*[tiab] OR leukaemi*[tiab] OR "leucocytos*"[tiab] OR thrombocyt*[tiab] OR hematopathy[tiab] OR hemopathy[tiab] OR "lymphatic disease*"[tiab] OR "malignant histiocytos*"[tiab] OR "malignant plasmacytoma*"[tiab] OR "mastocytoma"[tiab] OR "osteomyelopath*"[tiab] OR "aleukia"[tiab] OR ((blood[tiab] OR hematolog*[tiab] OR haematolog*[tiab] OR myeloproliferati*[tiab] OR myelodysplas*[tiab] OR myelofibro*[tiab] OR "bone marrow"[tiab] OR "leucocyt*"[tiab] OR "lymphatic*"[tiab] OR "lymphoid*"[tiab]) AND (cancer[sb] OR neoplasm*[tiab] OR disease*[tiab] OR disorder*[tiab] OR syndrom*[tiab] OR "malignanc*"[tiab] OR dyscras*[tiab]))	1,973,914
1	"Pulmonary Aspergillosis"[Mesh:NoExp] OR "Invasive Pulmonary Aspergillosis"[Mesh] OR "Lung aspergill*"[tiab] OR "Bronchial aspergill*"[tiab] OR "Pleural aspergill*"[tiab] OR "Pulmonary aspergill*"[tiab] OR "Pulmonary invasive aspergill*"[tiab] OR "Aspergillus fungal ball*"[tiab] OR "Aspergillus fungus ball*"[tiab] OR "Aspergillus mycetoma"[tiab] OR "Sinus aspergill*"[tiab] "invasive aspergillos*"[tiab] OR "disseminated aspergill*"[tiab] OR "invasive pulmonary aspergill*"[tiab] OR "systemic aspergill*"[tiab] OR (("Aspergillosis"[Mesh:NoExp] OR "aspergillos*"[tiab] OR "aspergillom*"[tiab]) AND ("lung*"[tiab] OR "bronch*"[tiab] OR "pleur*"[tiab] OR "pulmonar*"[tiab]))	10,09

EMBASE – 13-04-2021

No.	Query	Results
**#4**	**#1 AND #2 AND #3**	**1145**
#3	'galactomannan antigen'/exp OR 'galactomannan'/exp OR 'galactomannan test'/exp OR 'galactomannan':ti,ab,kw	4524
#2	'hematologic disease'/de OR 'blood dyscrasia'/exp OR 'bone marrow disease'/exp OR 'hematologic malignancy'/exp OR 'leukocyte disorder'/exp OR 'lymphatic system disease'/exp OR 'lymphoma*':ti,ab,kw OR 'myeloma*':ti,ab,kw OR 'myeloidos*':ti,ab,kw OR 'myelopath*':ti,ab,kw OR 'hodgkin*':ti,ab,kw OR 'nonhodgkin*':ti,ab,kw OR 'leukemi*':ti,ab,kw OR 'leukaemi*':ti,ab,kw OR 'leucocytos*':ti,ab,kw OR 'thrombocyt*':ti,ab,kw OR 'hematopathy':ti,ab,kw OR 'hemopathy':ti,ab,kw OR 'lymphatic disease*':ti,ab,kw OR 'malignant histiocytos*':ti,ab,kw OR 'malignant plasmacytoma*':ti,ab,kw OR 'mastocytoma':ti,ab,kw OR 'osteomedullopat*':ti,ab,kw OR 'osteomyelopath*':ti,ab,kw OR 'aleukia*':ti,ab,kw OR (('blood':ti,ab,kw OR 'hematolog*':ti,ab,kw OR	2663759

	'haematolog*':ti,ab,kw OR 'myeloproliferati*':ti,ab,kw OR 'myelodysplas*':ti,ab,kw OR 'myelofibro*':ti,ab,kw OR 'bone marrow':ti,ab,kw OR 'leucocyt*':ti,ab,kw OR 'lymhatic*':ti,ab,kw OR 'lymhoid*':ti,ab,kw) AND ('cancer':ti,ab,kw OR 'neoplasm*':ti,ab,kw OR 'disease*':ti,ab,kw OR 'disorder*':ti,ab,kw OR 'syndrom*':ti,ab,kw OR 'malignanc*':ti,ab,kw OR 'discras*':ti,ab,kw))	
#1	'lung aspergillosis'/exp OR 'invasive aspergillosis'/exp OR 'lung aspergill*':ti,ab,kw OR 'bronchial aspergill*':ti,ab,kw OR 'bronchus aspergill*':ti,ab,kw OR 'pleura aspergill*':ti,ab,kw OR 'pleural aspergill*':ti,ab,kw OR 'pulmonary aspergill*':ti,ab,kw OR 'lung invasive aspergill*':ti,ab,kw OR 'bronchial invasive aspergill*':ti,ab,kw OR 'bronchus invasive aspergill*':ti,ab,kw OR 'pleura invasive aspergill*':ti,ab,kw OR 'pleural invasive aspergill*':ti,ab,kw OR 'pulmonary invasive aspergill*':ti,ab,kw OR 'aspergillus fungal ball*':ti,ab,kw OR 'aspergillus fungus ball*':ti,ab,kw OR 'aspergillus mycetoma':ti,ab,kw OR 'brongopulmonary aspergill*':ti,ab,kw OR 'sinus aspergill*':ti,ab,kw OR 'invasive aspergillos*':ti,ab,kw OR 'disseminated aspergill*':ti,ab,kw OR 'invasive pulmonary aspergill*':ti,ab,kw OR 'systemic aspergill*':ti,ab,kw OR (('aspergillosis'/de OR 'aspergilloma'/exp OR 'aspergillos*':ti,ab,kw OR 'aspergillom*':ti,ab,kw) AND ('lung*':ti,ab,kw OR 'bronch*':ti,ab,kw OR 'pleur*':ti,ab,kw OR 'pulmonar*':ti,ab,kw))	21435

360 conference abstracts

Cochrane – 13-04-2021

(galactomannan):ti,ab,kw	83
MeSH descriptor: [Hematologic Diseases] explode all trees	14716
MeSH descriptor: [Hematologic Neoplasms] explode all trees	582
MeSH descriptor: [Leukemia] explode all trees	4751
MeSH descriptor: [Lymphoma] explode all trees	3281
M<br>eSH descriptor: [Multiple Myeloma] explode all trees	1633
(lymphoma* OR myeloma* OR "myeloidos*" OR "myelopathy*" OR hodgkin* OR nonhodgkin* OR leukemi* OR leukaemi* OR "leucocytos*" OR thrombocyt* OR hematopathy OR hemopathy OR "lymphatic disease*" OR "malignant histiocytos*" OR "malignant plasmacytoma*" OR "mastocytoma" OR "osteomedullopat*" OR "osteomyelopath*" OR "aleukia"):ti, ab, kw	43573
(blood OR hematolog* OR haematolog* OR myeloproliferati* OR myelodysplas* OR myelofibro* OR "bone marrow" OR "leucocyt*" OR "lymphatic*" OR "lymphoid*"):ti,ab,kw	393944
(cancer* OR neoplasm* OR disease* OR disorder* OR syndrom* OR "malignanc*" OR dyscras*):ti, ab, kw	703955
#2 OR #3 OR #4 OR #5 OR #6 OR #7 OR (#8 AND #9)	208594
MeSH descriptor: [Pulmonary Aspergillosis] this term only	29
MeSH descriptor: [Invasive Pulmonary Aspergillosis] explode all trees	15
('Lung aspergill*' OR 'Bronchial aspergill*' OR 'Bronchus aspergill*' OR 'Pleura aspergill*' OR 'Pleural aspergill*' OR 'Pulmonary aspergill*' OR 'Lung invasive aspergill*' OR 'Bronchial invasive aspergill*' OR 'Bronchus invasive aspergill*' OR 'Pleura invasive aspergill*' OR 'Pleural invasive aspergill*' OR 'Pulmonary invasive aspergill*' OR 'Aspergillus fungal ball*' OR 'Aspergillus fungus ball*' OR 'Aspergillus mycetoma' OR 'Brongopulmonary aspergill*' OR 'Sinus aspergill*' OR 'invasive aspergillos*' OR 'Disseminated aspergill*' OR 'Invasive pulmonary aspergill*' OR 'Systemic aspergill*'):ti,ab,kw	511
MeSH descriptor: [Aspergillosis] this term only	148
(aspergillos* OR aspergillom*):ti.ab,kw	536
(lung* OR bronch* OR pleur* OR pulmonar*):ti,ab,kw	120702
#11 OR #12 OR #13 OR ((#14 OR #15) AND (#16))	545
**#1 AND #10 AND #17**	**32**

29 trials

Figure S1 – Article Selection Process[15]

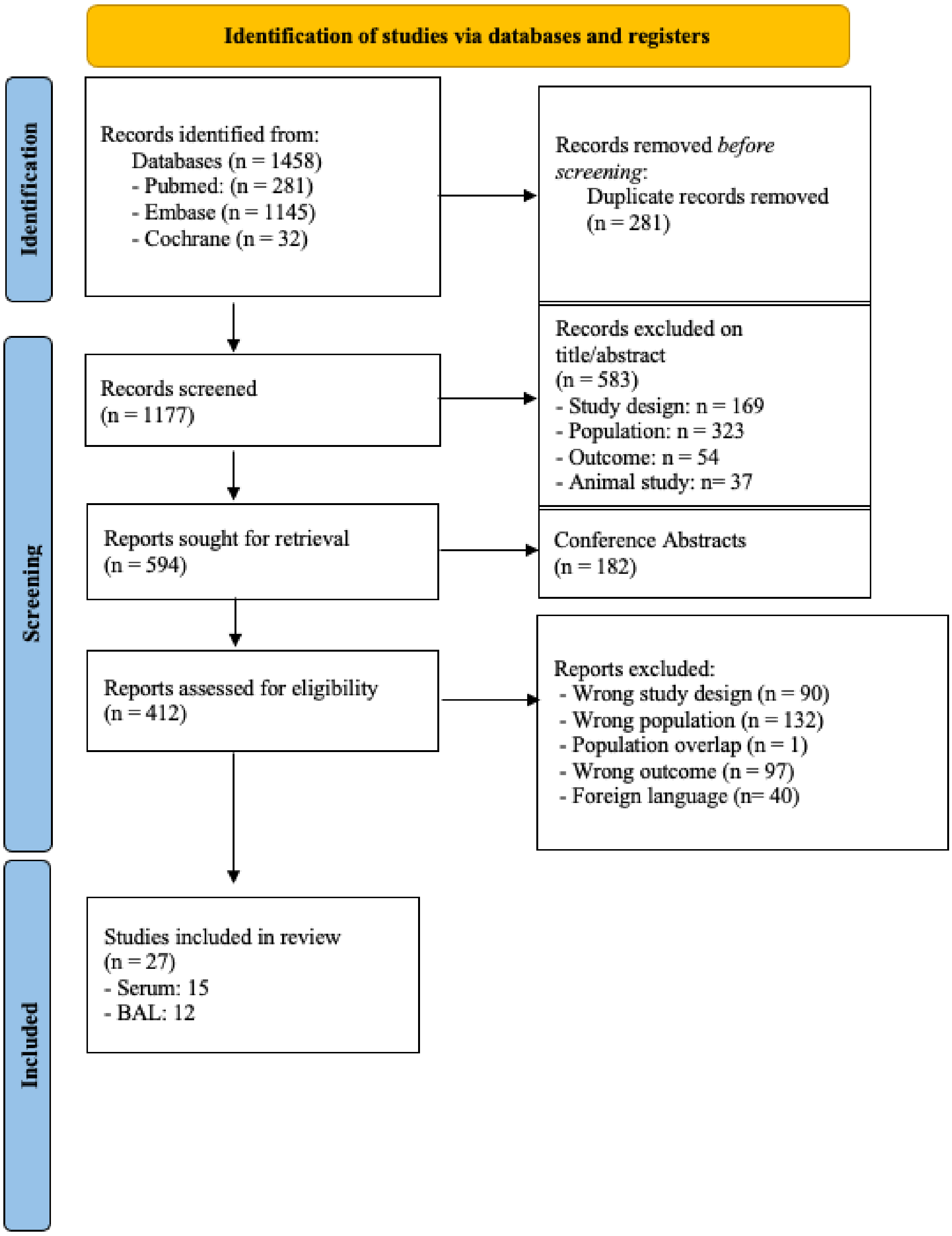

Table S1 – Serum Galactomannan Study Characteristics of studies included in Meta-Analysis

Study	Year of publication	Country	Data collection	Study design	Sampling method	Patient Population	Mean age: year (range)	Sample size (N=)**	proven/ probable	Number of possible	Diagnostic criteria	GM excluded as mycologic criteria	Cut-off used
Barnes (2)	2013	UK	Prospective	Cohort	Consecutive	* with risk for IPA	NA	549	53	23	EORTC/MSG 2008	No	2x≥0.5 + ≥0.5
Aslan (17)	2015	Turkey	Prospective	Cohort	Unclear	* suspected of IPA	NA	161	18	60	EORTC/MSG 2008	Unclear	≥0.5
Becker(a) (18)	2003	Netherlands	Prospective	Cohort	Unclear	* with risk for IPA	49 (18–79)	75	13	18	EORTC/MSG 2002	Yes	2x≥1.0
Boch (20)	2016	Germany	Prospective	Cohort	Unclear	* with risk for IPA	NA	82	20	33	EORTC/MSG 2008	Yes	≥0.5
Bölük (21)	2016	Turkey	Retrospective	Cohort	Unclear	* with risk for IPA	NA	70	40	17	EORTC/MSG 2008	No	2x≥0.5
Furfaro (23)	2018	Italy	Retrospective	Cohort	Consecutive	* with risk for IPA	58 (20-98)	167	20	0	EORTC/MSG 2008	No	≥0.5
Heldt (24)	2018	Austria	Prospective	Cohort	Consecutive	* suspected of IPA	NA (26-82)	106	11	32	EORTC/MSG 2008	Yes	≥0.5
Jin (28)	2013	China	Prospective	Cohort	Consecutive	* with risk for IPA	39 (20-76)	378	143	45	EORTC/MSG 2008	Yes	2x≥0.5
Mercier (29)	2020	Belgium	Prospective	Cohort	Consecutive	* with risk for IPA	60 (NA)	229	34	0	EORTC/MSG 2020	Yes	2x≥0.5 or ≥0.8
Pazos (32)	2005	Spain	Retrospective	Cohort	Not consecutive	* with risk for IPA	44 (18-70)	40	8	3	EORTC/MSG 2002	Yes	2x≥1.5
Persat (33)	2008	France	Retrospective	Case-control	Unclear	* with risk for IPA	NA	170	70	0	EORTC/MSG 2002	Unclear	≥0.5
Rogers(a) (34)	2013	Ireland	Prospective	Cohort	Consecutive	* with risk for IPA	NA	146	15	11	EORTC/MSG 2008	No	2x≥0.5 + ≥0.5
Rogers(b) (34)	2013	Ireland	Prospective	Cohort	Consecutive	* with risk for IPA	NA	132	30	19	EORTC/MSG 2008	No	2x≥0.5 + ≥0.5
Suarez (36)	2008	France	Prospective	Cohort	Consecutive	* with risk for IPA	NA	138	15	2	EORTC/MSG 2002	Yes	2x≥0.5 + ≥0.5
White (38)	2013	UK	Retrospective	Case-control	Not consecutive	* suspected of IPA	NA	103	22	22	EORTC/MSG 2008	No	2x≥0.5 + ≥0.5
Yoo (39)	2007	Korea	Prospective	Cohort	Consecutive	* suspected of IPA	NA	22	7	15	EORTC/MSG 2002	Unclear	2x≥0.5

*Adult hematological malignancy patients
** Number of patients or samples used for our analysis
NA = Not Available

Table S2 – BAL Galactomannan Study Characteristics of studies included in Meta-Analysis

Study	Year of publication	Country	Data collection	Study design	Sampling method	Patient Population	Mean age: year (range)	Sample size (N=) **	Number of proven/ probable	Number of possible	Diagnostic standard	GM excluded as mycologic criteria	Cut-off used
Becker(a)(18)	2003	Netherlands	Prospective	Cohort	Unclear	* suspected of IPA	NA	25	7	2	EORTC/MSG 2002	Yes	>1.0
Becker(b)(18)	2003	Netherlands	Prospective	Cohort	Unclear	* suspected of IPA	47 (18-74)	45	12	12	EORTC/MSG 2002	Yes	>1.0
Bergeron (19)	2010	France	Retrospective	Cohort	Consecutive	* suspected of IPA	45 (NA)	101	29	4	EORTC/MSG 2008	Yes	≥0.5
Boch (20)	2016	Germany	Prospective	Cohort	Unclear	* suspected of IPA	NA	82	20	33	EORTC/MSG 2008	Yes	≥0.5
Frealle (22)	2009	France	Retrospective	Cohort	Unclear	* suspected of IPA	NA	57	25	0	EORTC/MSG 2008	No	≥1.0
Heldt (24)	2018	Austria	Prospective	Cohort	Unclear	* suspected of IPA	58 (26-82)	106	11	32	EORTC/MSG 2008	Unclear	≥0.5
Heng (25)	2014	Australia	Retrospective	Cohort	Consecutive	* suspected of IPA	57 (18-80)	116	18	50	EORTC/MSG 2008	Yes	≥0.5, ≥0.8, ≥1.0
Hoenigl (26)	2018	Australia	Prospective	Cohort	Convenience series	* suspected of IPA	NA	14	9	0	EORTC/MSG 2008	Unclear	≥1.0
Hsu (27)	2010	Singapore	Prospective	Case-control	Unclear	* suspected of IPA	NA	26	9	0	EORTC/MSG 2008	Yes	≥0.5
Mercier (30)	2019	Belgium/ Netherlands	Retrospective	Cohort	Consecutive	* suspected of IPA	63 (NA)	247	33	44	EORTC/MSG 2008	Yes	≥1.0
Nguyen (31)	2011	US	Retrospective	Cohort	Consecutive	* suspected of IPA	63 (NA)	89	15	32	EORTC/MSG 2008	Yes	≥0.5, ≥0.85, ≥1.0
Sanguinetti (35)	2003	Italy	Prospective	Cohort	Consecutive	* suspected of IPA	60.3 (39–77)	44	20	0	EORTC/MSG 2002	No	≥1.5
Wehrle-Wiel (37)	2018	Switzerland	Prospective	Cohort	Unclear	* suspected of IPA	57 (21-87)	138	33	29	EORTC/MSG 2008	Yes	≥0.5, ≥1.0

*Adult hematological malignancy patients
** Number of patients or samples used for our analysis
NA = Not Available

Figure S2 – Detailed methodological quality assessment of serum studies included in meta-analysis using the QUADAS-2 tool

	Risk of Bias				Applicability Concerns		
	Patient Selection	Index Test	Reference Standard	Flow and Timing	Patient Selection	Index Test	Reference Standard
Aslan (2015)	+	+	?	+	+	+	+
Barnes (2013)	+	+	-	+	+	+	+
Becker(a) (2003)	+	+	+	-	+	+	+
Boch (2016)	-	+	-	-	?	+	+
Bölük (2018)	+	+	?	+	+	+	+
Furfaro (2018)	-	+	-	+	+	+	+
Heldt (2018)	+	+	+	-	+	+	+
Jin (2013)	+	+	+	+	+	+	+
Mercier (2020)	+	+	+	+	-	+	+
Pazos (2005)	-	+	+	+	+	+	+
Persat (2008)	-	+	?	+	+	+	+
Rogers(a) (2013)	+	+	-	+	+	+	+
Rogers(b) (2013)	+	+	-	+	+	+	+
Suarez (2008)	+	+	+	+	+	+	+
White (2013)	-	+	-	+	+	+	+
Yoo (2007)	+	+	?	+	?	+	+

- High ? Unclear + Low

Figure S3 – Overall methodological quality assessment of serum studies included in meta-analysis using the QUADAS-2 tool

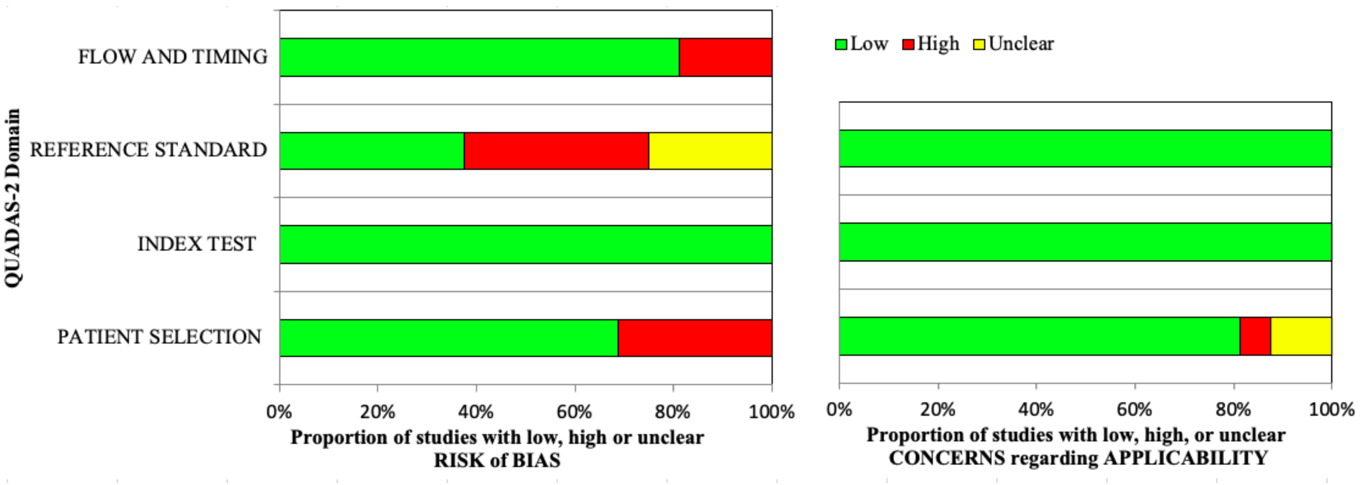

Figure S4 – Detailed methodological quality assessment of BAL studies included in meta-analysis using the QUADAS-2 tool

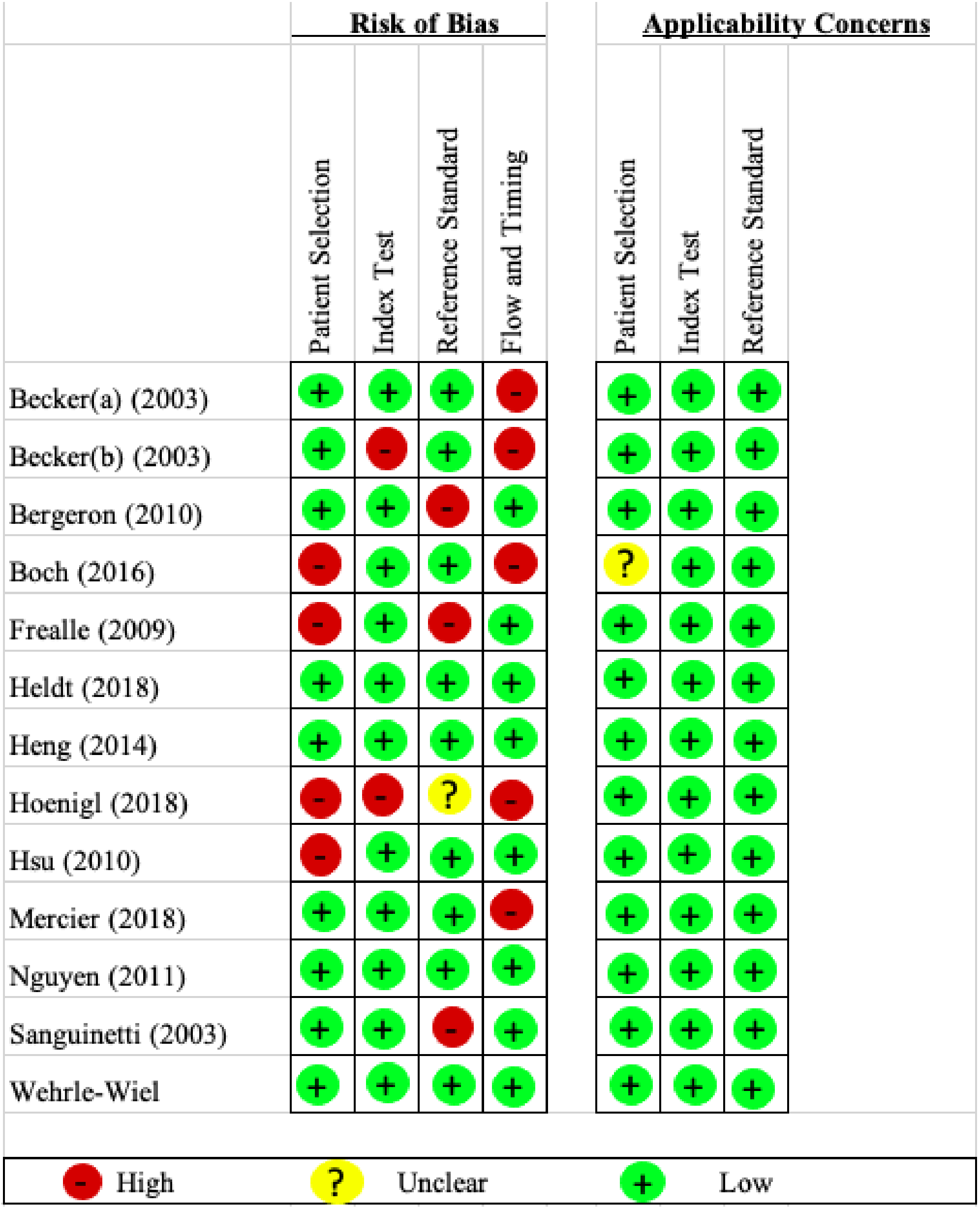

Figure S5 – Overall methodological quality assessment of BAL studies included in meta-analysis using the QUADAS-2 tool

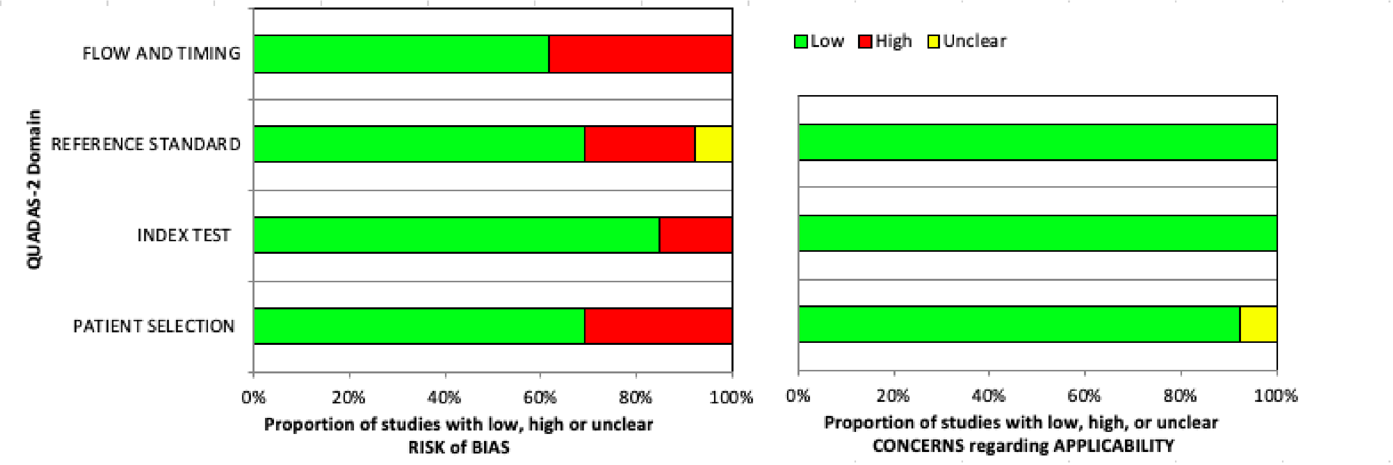

Table S3 – Serum Galactomannan Study Results cut-off ≥0.5

Author (year)	Diagnostic data cut-off ≥0.5											
	Proven/probable vs no IA						Proven/probable/possible vs no IA					
	TP	FP	FN	TN	Sensitivity [95%CI]	Specificity [95%CI]	TP	FP	FN	TN	Sensitivity [95%CI]	Specificity [95%CI]
Aslan (2015)[a*]	18	8	0	75	100.00 [81.47-100.00]	90.36 [81.89-95.75]	18	8	60	75	23.08 [14.29-34.00]	90.36 [81.89-95.75]
Barnes (2013)	51	110	2	363	96.23 [87.02-99.54]	76.74 [72.67-80.48]	51	110	25	363	67.11 [55.37-77.46]	76.74 [72.67-80.48]
Boch (2016)	7	0	13	29	35.00 [15.39-59.22]	100.00 [88.06-100.00]	NA	NA	NA	NA	NA	NA
Furfaro (2018)	19	14	1	133	95.00 [75.13-99.87]	90.48 [84.54-94.69]	NA	NA	NA	NA	NA	NA
Heldt (2018)[*a]	NA	NA	NA	NA	NA	NA	2	2	41	61	4.65 [0.57-15.81]	96.83 [89.00-99.61]
Persat (2008)	39	21	21	79	65.00 [51.60-76.87]	79.00 [69.71-86.51]	NA	NA	NA	NA	NA	NA
Rogers(a)(2013)	15	28	0	92	100.00 [78.20-100.00]	76.67 [68.07-83.90]	NA	NA	NA	NA	NA	NA
Rogers(b)(2013)	28	25	2	57	93.33 [77.93-99.18]	69.51 [58.36-79.20]	NA	NA	NA	NA	NA	NA
Suarez (2008)	15	19	0	102	100.00 [78.20-100.00]	84.30 [76.57-90.27]	16	19	1	102	94.12 [71.31-99.85]	84.30 [76.57-90.27]
White (2013)[a]	17	11	5	48	77.27 [54.63-92.18]	81.36 [69.09-90.31]	17	11	27	48	38.64 [24.36-54.50]	81.36 [69.09-90.31]

Table S4 – Serum Galactomannan Study Results cut-off 2x≥0.5

Author (year)	Diagnostic data cut-off 2x≥0.5											
	Proven/probable vs no IA						Proven/probable/possible vs no IA					
	TP	FP	FN	TN	Sensitivity [95%CI]	Specificity [95%CI]	TP	FP	FN	TN	Sensitivity [95%CI]	Specificity [95%CI]
Barnes (2013)	33	39	20	434	62.26 [47.89-75.21]	91.75[88.90-94.07]	33	39	43	434	43.42 [32.08-55.29]	91.75 [88.90-94.07]
Bölük (2016)	36	0	4	13	90.00 [76.34-97.21]	100.00 [75.29-100.00]	36	0	21	13	63.16 [49.34-75.55]	100.00 [75.29-100.00]
Jin (2013)[a]	69	5	74	185	48.25 [39.82-56.75]	97.37 [93.97-99.14]	NA	NA	NA	NA	NA	NA
Mercier (2020)[o]	11	2	23	186	32.35 [17.39-50.53]	98.94 [96.21-99.87]	NA	NA	NA	NA	NA	NA
Rogers(a)(2013)	9	10	6	110	60.00 [32.29-83.66]	91.67 [85.21-95.93]	NA	NA	NA	NA	NA	NA
Rogers(b)(2013)	14	9	16	74	46.67 [28.34-65.67]	89.16 [80.41-94.92]	NA	NA	NA	NA	NA	NA
Suarez (2008)	15	5	0	116	100.00 [78.20-100.00]	95.87 [90.62-98.64]	15	5	2	116	88.24 [63.56-98.54]	95.87 [90.62-98.64]
White (2013)	15	5	7	54	68.18 [45.13-86.14]	91.53 [81.32-97.19]	15	5	29	54	34.09 [20.49-49.92]	91.53 [81.32-97.19]
Yoo (2007)[*a]	5	1	2	55	71.43 [29.04-96.33]	98.21 [90.45-99.95]	10	1	12	55	45.45 [24.39-67.79]	98.21 [90.45-99.95]

Table S5 – Serum Galactomannan Study Results cut-off 2x≥1.0

Author (year)	Diagnostic data cut-off 2x≥1.0											
	Proven/probable vs no IA						Proven/probable/possible vs no IA					
	TP	FP	FN	TN	Sensitivity [95%CI]	Specificity [95%CI]	TP	FP	FN	TN	Sensitivity [95%CI]	Specificity [95%CI]
Becker(a)	6	3	7	41	46.15 [19.22-74.87]	93.18 [81.34-98.57]	10	3	21	41	32.26 [16.68-51.37]	93.18 [81.34-98.57]

Table S6 – Serum Galactomannan Study Results cut-off 2x≥1.5

Author (year)	Diagnostic data cut-off 2x≥1.5											
	Proven/probable vs no IA						Proven/probable/possible vs no IA					
	TP	FP	FN	TN	Sensitivity [95%CI]	Specificity [95%CI]	TP	FP	FN	TN	Sensitivity [95%CI]	Specificity [95%CI]
Pazos (2005)	7	3	1	26	87.50 [47.35-99.69]	89.66 [72.65-97.81]	8	3	3	26	72.73 [39.03-93.98]	89.66 [72.65-97.81]

*There were no proven patients in this population

[a] Population had higher risk for IPA

[o] Used a cut-off value of 2x≥0.5 or 1x≥0.8

NA=Not Available

TP=True Positive

FP=False Positive

FN=False Negative

TN=True Negative

CI=Confidence Interval

Figure S6 – Forest plot of diagnostic power of sensitivity serum galactomannan as a diagnostic assay for proven/probable IPA versus no-IPA.

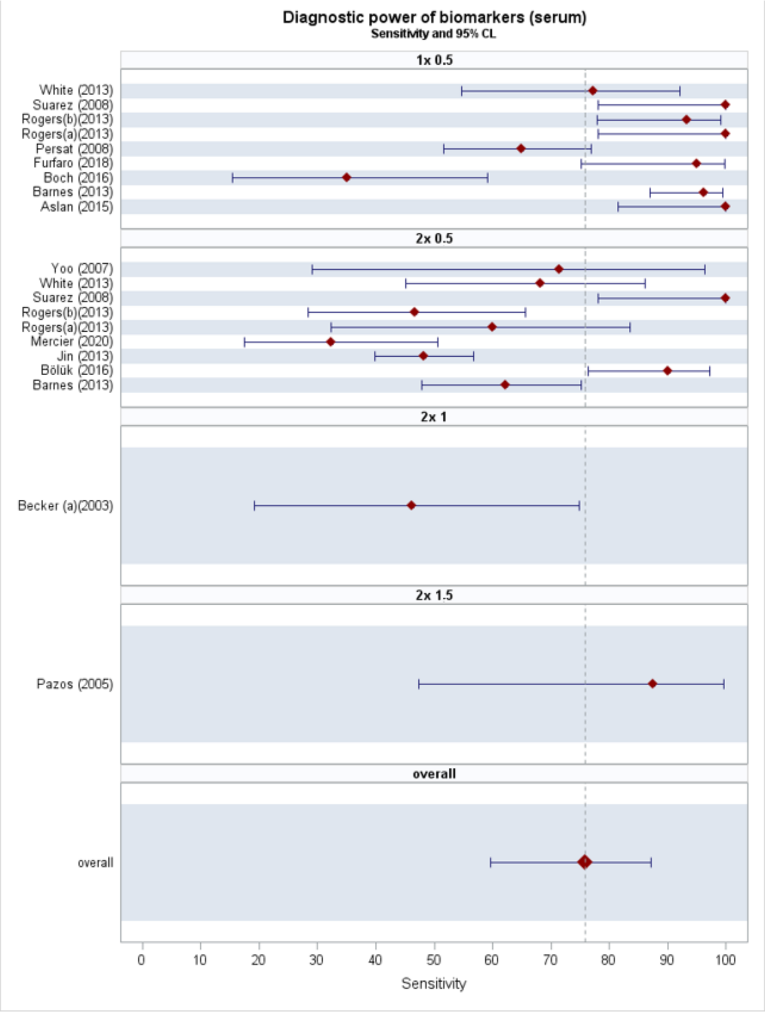

Figure S7 – Forest plot of diagnostic power of specificity serum galactomannan as a diagnostic assay for proven/probable IPA versus no-IPA.

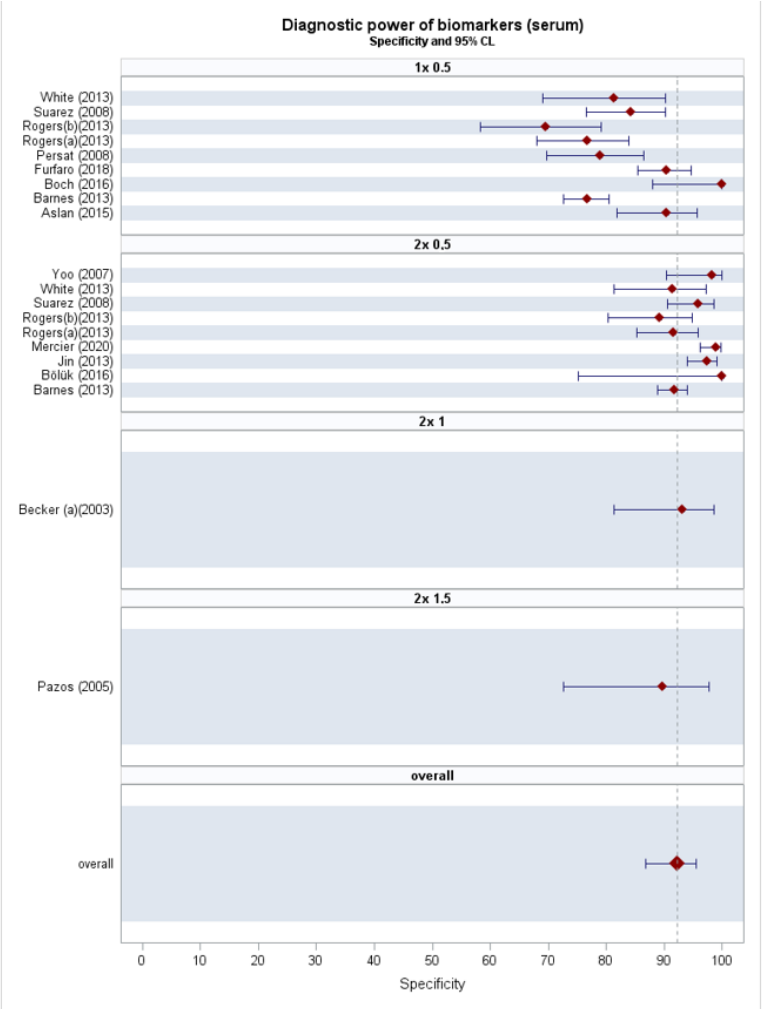

Figure S8 – Forest plot of diagnostic power of sensitivity serum galactomannan as a diagnostic assay for proven/probable/possible IPA versus no-IPA.

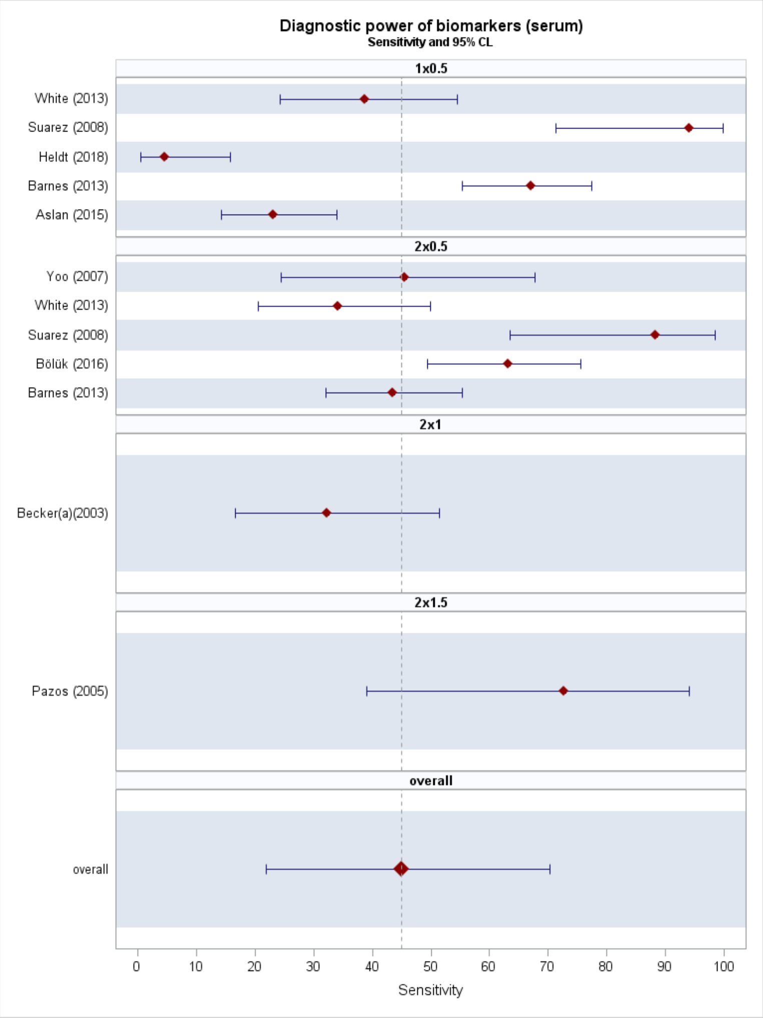

Figure S9 – Forest plot of diagnostic power of specificity serum galactomannan as a diagnostic assay for proven/probable/possible IPA versus no-IPA.

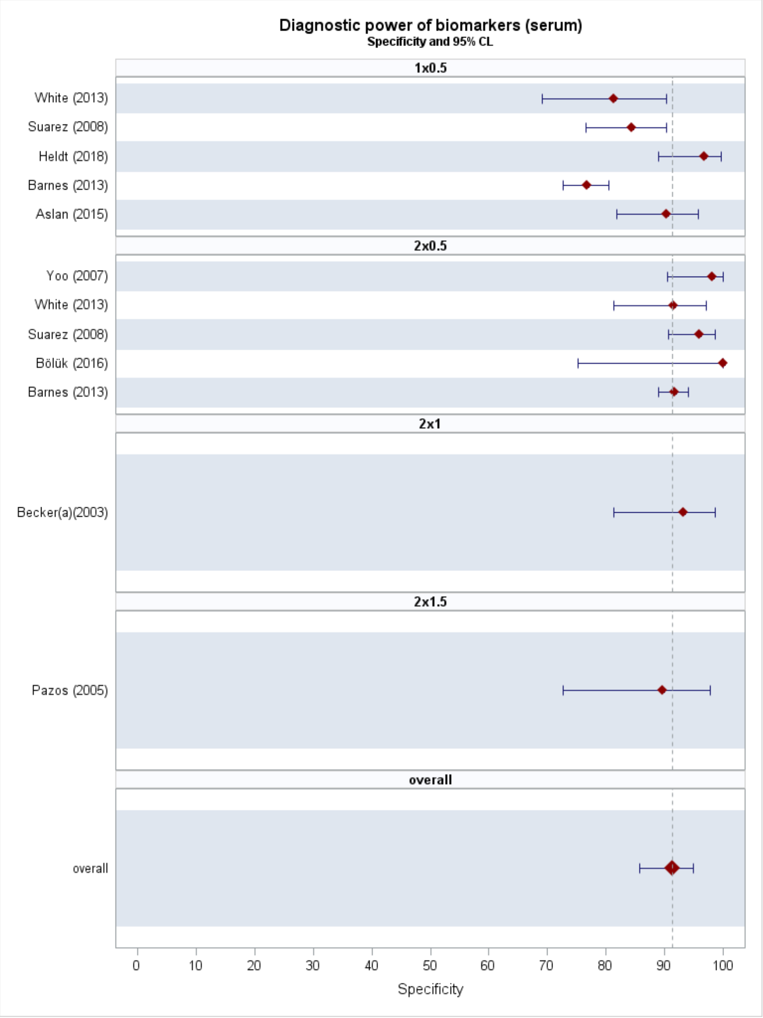

Figure S10 – Forest plot of diagnostic power of sensitivity serum galactomannan as a diagnostic assay per cut-off proven/probable IPA versus no-IPA.

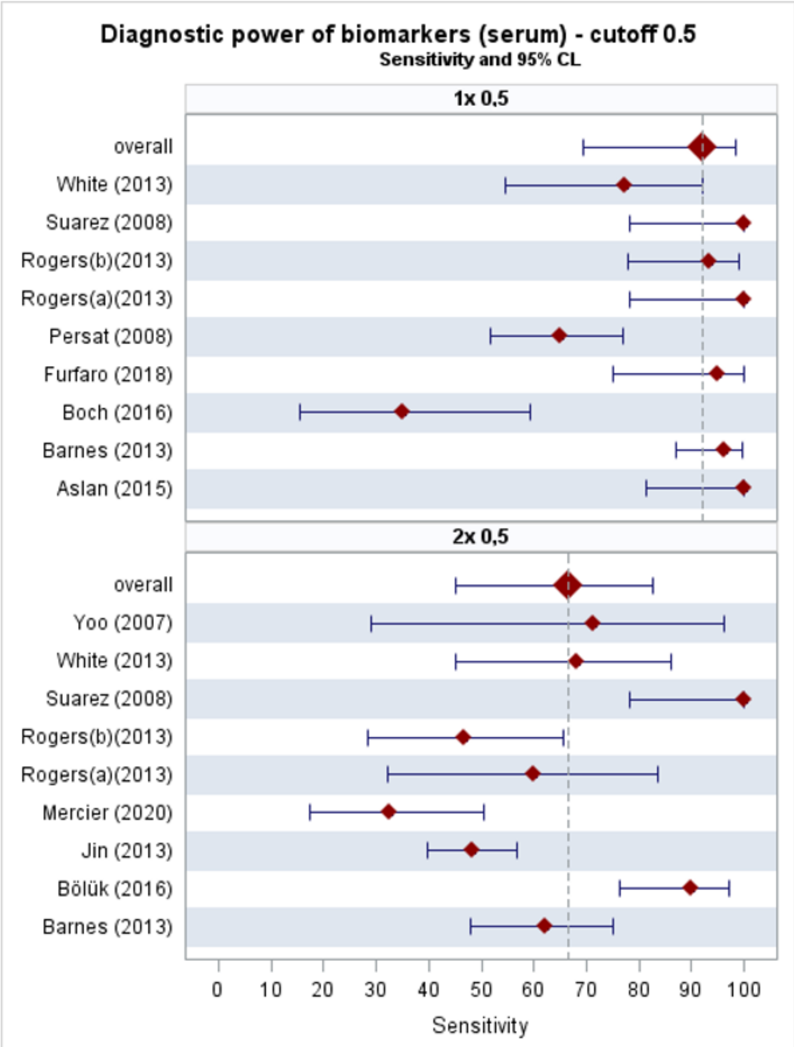

Figure S11 – Forest plot of diagnostic power of specificity serum galactomannan as a diagnostic assay per cut-off proven/probable IPA versus no-IPA.

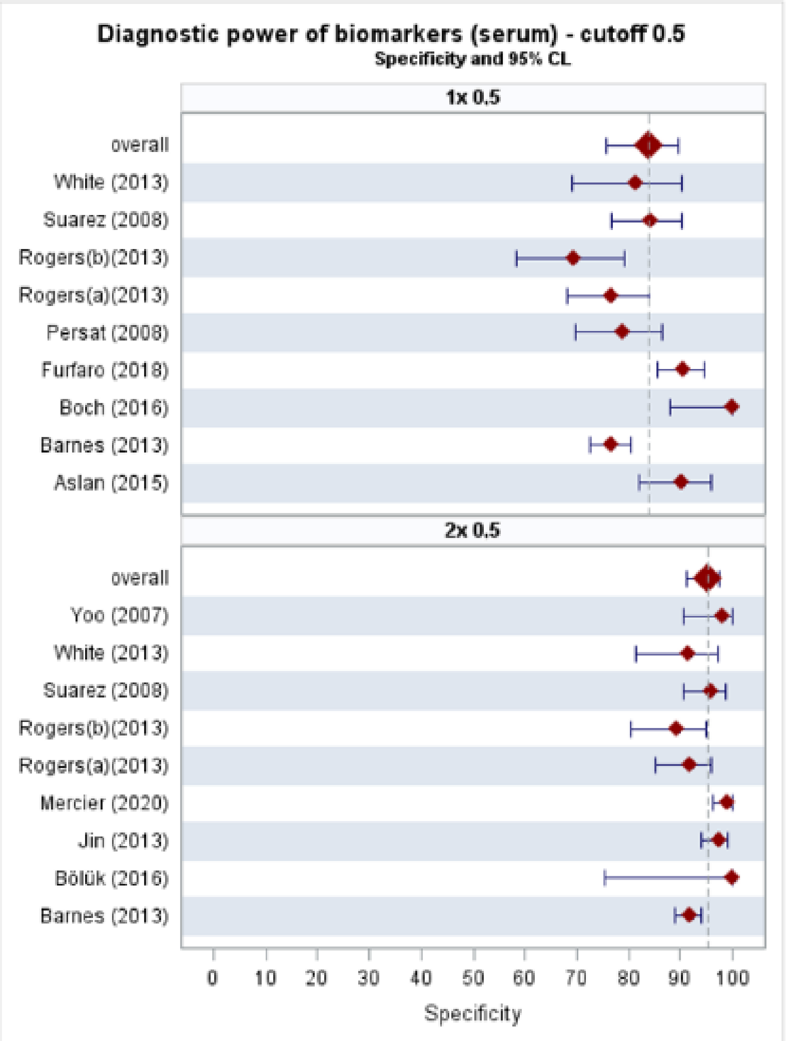

Table S7 - Pooled results of the included studies for serum GM for proven/probable IPA vs. no IPA

Cut-off value	Nr. of studies	Sensitivity [95% CI]	Specificity [95% CI]
**0.5**	9	0.92 [0.69-0.98]	0.84 [0.76-0.90]
**2x0.5**	9	0.66 [0.45-0.83]	0.95 [0.91-0.98]
**1.0**	1	0.46 [0.19-0.75]	0.93 [0.81-0.99]
**1.5**	1	0.88 [0.47-1.00]	0.90 [0.73-0.98]

Test difference in using 1x or 2x
Sensitivity: pvalue=0,0036
Specificity: pvalue=0,0004

Table S8 – Results subanalysis excluding studies with higher pre-test probability of the included studies for serum GM for proven/probable IPA vs. no IPA

Cut-off value	Nr. of studies	Sensitivity [95% CI]	Specificity [95% CI]
**0.5**	8	0.90 [0.64-0.98]	0.83 [0.74-0.89]
**2x0.5**	8	0.69 [0.40-0.88]	0.94 [0.89-0.97]
**Overall**	8	0.75 [0.57-0.87]	0.91 [0.84-0.95]

Test difference in using 1x or 2x
Sensitivity: pvalue=0,0041
Specificity: pvalue=0,0012

Figure S12 – Forest plot of diagnostic power of sensitivity serum galactomannan as a diagnostic assay per cut-off proven/probable/possible IPA versus no-IPA.

Figure S13 – Forest plot of diagnostic power of specificity serum galactomannan as a diagnostic assay per cut-off proven/probable/possible IPA versus no-IPA.

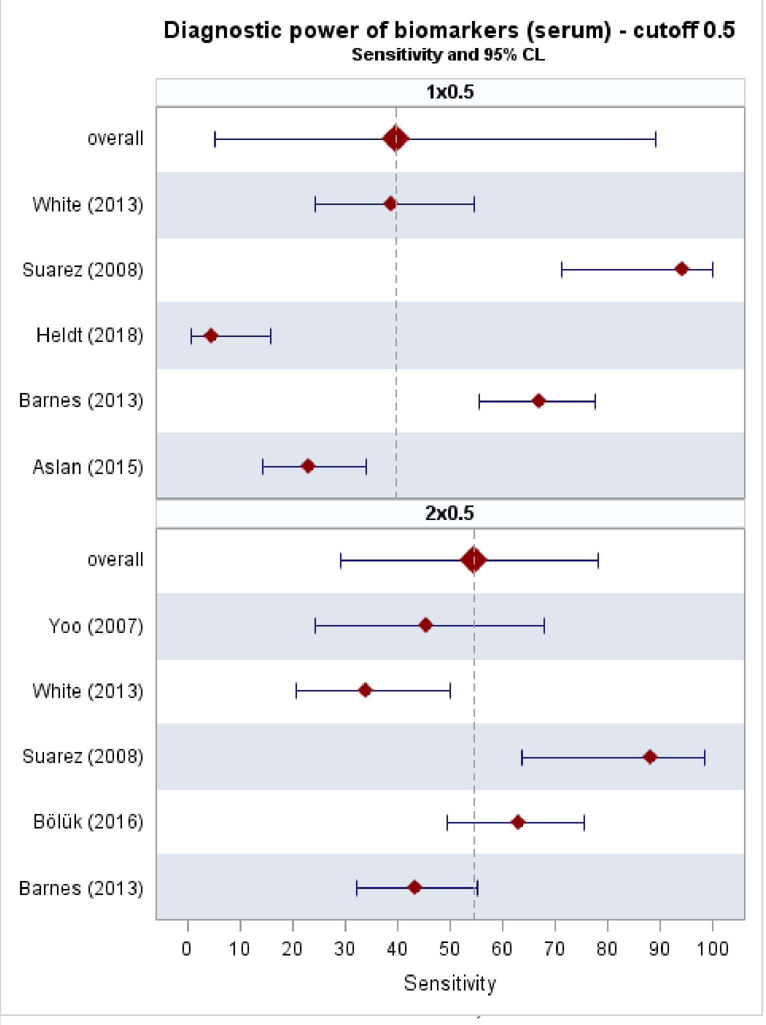

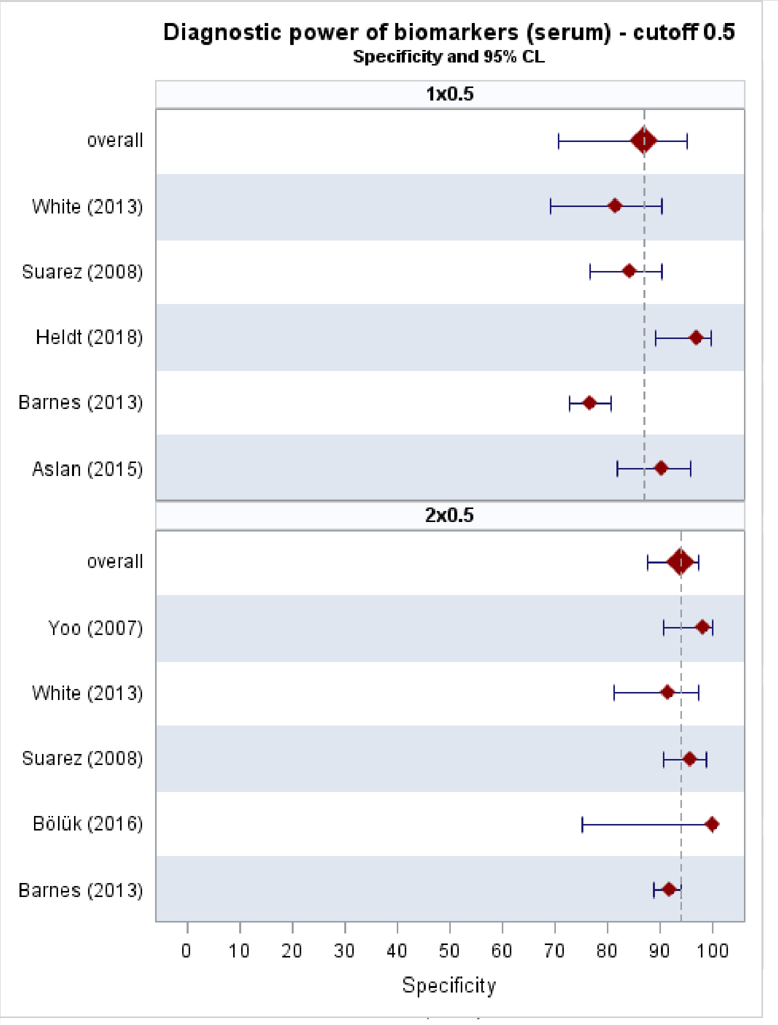

Table S9 – Pooled results of the included studies for serum GM for proven/probable/possible IPA vs. no IPA

Cut-off value	Nr. of studies	Sensitivity [95% CI]	Specificity [95% CI]
**0.5**	5	0.40 [0.05-0.89]	0.87 [0.71-0.95]
**2x0.5**	5	0.55 [0.29-0.78]	0.94 [0.87-0.97]
**1.0**	1	0.32 [0.17-0.51]	0.93 [0.81-0.99]
**1.5**	1	0.73 [0.39-0.94]	0.90 [0.73-0.98]

Test difference in using 1x or 2x
Sensitivity: pvalue=0.2617
Specificity: pvalue=0.0367
-> less values (also big spread) -> higher uncertainty

Table S10 – Results subanalysis excluding studies with higher pre-test probability of the included studies for serum GM for proven/probable IPA vs. no IPA

Cut-off value	Nr. of studies	Sensitivity [95% CI]	Specificity [95% CI]
**0.5**	3	0.72 [0.01-1.0]	0.79 [0.39-0.95]
**2x0.5**	4	0.57 [0.16-0.90]	0.93 [0.83-0.98]
**Overall**	4	0.60 [0.33-0.82]	0.87 [0.80-0.92]

Table S11 – BAL Galactomannan Study Results cut-off ≥0.5

Author (year)	Diagnostic data cut-off ≥0.5											
	Proven/probable vs no IA						Proven/probable/possible vs no IA					
	TP	FP	FN	TN	Sensitivity [95%CI]	Specificity [95%CI]	TP	FP	FN	TN	Sensitivity [95%CI]	Specificity [95%CI]
Bergeron (2010)	17	3	12	65	58.62 [38.94-76.48]	95.59 [87.64-99.08]	19	3	14	65	57.58 [39.22-74.52]	95.59 [87.64-99.08]
Boch (2016)	16	1	4	28	80.00 [56.34-94.27]	96.55 [82.24-99.91]	NA	NA	NA	NA	NA	NA
Heldt (2018)	NA	NA	NA	NA	NA	NA	10	5	33	58	23.26 [11.76-38.63]	92.06 [82.44-97.37]
Heng (2014)	11	6	7	36	61.11 [35.75-82.70]	85.71 [71.46-94.57]	NA	NA	NA	NA	NA	NA
Hsu (2010)	9	3	0	14	100.00 [66.37-100.00]	82.35 [56.57-96.20]	NA	NA	NA	NA	NA	NA
Nguyen (2011)	11	4	4	33	73.33 [44.90-92.21]	89.19 [74.58-96.97]	22	4	25	33	46.81 [32.11-61.92]	89.19 [74.58-96.97]
Wehrle-Wiel (2018)	28	24	5	81	84.85 [68.10-94.89]	77.14 [67.93-84.77]	NA	NA	NA	NA	NA	NA

Table S12 – BAL Galactomannan Study Results cut-off ≥0.8/0.85

Author (year)	Diagnostic data cut-off ≥0.8/0.85											
	Proven/probable vs no IA						Proven/probable/possible vs no IA					
	TP	FP	FN	TN	Sensitivity [95%CI]	Specificity [95%CI]	TP	FP	FN	TN	Sensitivity [95%CI]	Specificity [95%CI]
Heng (2014)*	11	3	7	39	61.11 [35.75-82.70]	92.86 [80.52-98.50]	21	3	47	39	30.88 [20.24-43.26]	92.86 [80.52-98.50]
Nguyen (2011)**	10	2	5	35	66.67 [38.38-88.18]	94.59 [81.81-99.34]	19	2	28	35	40.43 [26.37-55.73]	94.59 [81.81-99.34]

Table S13 – BAL Galactomannan Study Results cut-off ≥1.0

Author (year)	Diagnostic data cut-off ≥1.0											
	Proven/probable vs no IA						Proven/probable/possible vs no IA					
	TP	FP	FN	TN	Sensitivity [95%CI]	Specificity [95%CI]	TP	FP	FN	TN	Sensitivity [95%CI]	Specificity [95%CI]
Becker(a)(2003)	7	1	0	17	100.00 [59.04-100.00]	94.44 [72.71-99.86]	8	0	1	16	88.89 [51.75-99.72]	100.00 [79.41-100.00]
Becker(b)(2003)	11	0	1	21	91.67 [61.52-99.79]	100.00 [83.89-100.00]	16	0	8	21	66.67 [44.68-84.37]	100.00 [83.89-100.00]
Frealle (2009)	18	0	7	32	72.00 [50.61-87.93]	100.00 [89.11-100.00]	NA	NA	NA	NA	NA	NA
Heng (2014)	10	3	8	39	55.56 [30.76-78.47]	92.86 [80.52-98.50]	NA	NA	NA	NA	NA	NA
Hoenigl (2018)	8	0	1	5	88.89 [51.75-99.72]	100.00 [47.82-100.00]	NA	NA	NA	NA	NA	NA
Mercier (2018)	28	5	5	119	84.85 [68.10-94.89]	95.97 [90.84-98.68]	NA	NA	NA	NA	NA	NA
Nguyen (2011)	8	2	7	35	53.33 [26.59-78.73]	94.59 [81.81-99.34]	15	2	32	35	31.91 [19.09-47.12]	94.59 [81.81-99.34]
Wehrle-Wiel (2018)	17	9	16	96	51.52 [33.54-69.20]	91.43 [84.35-96.01]	NA	NA	NA	NA	NA	NA

Table S14 – BAL Galactomannan Study Results cut-off ≥1.5

Author (year)	Diagnostic data cut-off ≥1.5											
	Proven/probable vs no IA						Proven/probable/possible vs no IA					
	TP	FP	FN	TN	Sensitivity [95%CI]	Specificity [95%CI]	TP	FP	FN	TN	Sensitivity [95%CI]	Specificity [95%CI]
Sanguinetti (2003)	20	0	0	24	100.00 [83.16-100.00]	100.00 [85.75-100.00]	NA	NA	NA	NA	NA	NA

*≥0.8

**≥0.85

NA=Not Available
TP=True Positive
FP=False Positive
FN=False Negative
TN=True Negative
CI=Confidence Interval

Figure S14 – Forest plot of diagnostic power of sensitivity BAL galactomannan as a diagnostic assay for proven/probable IPA versus no-IPA.

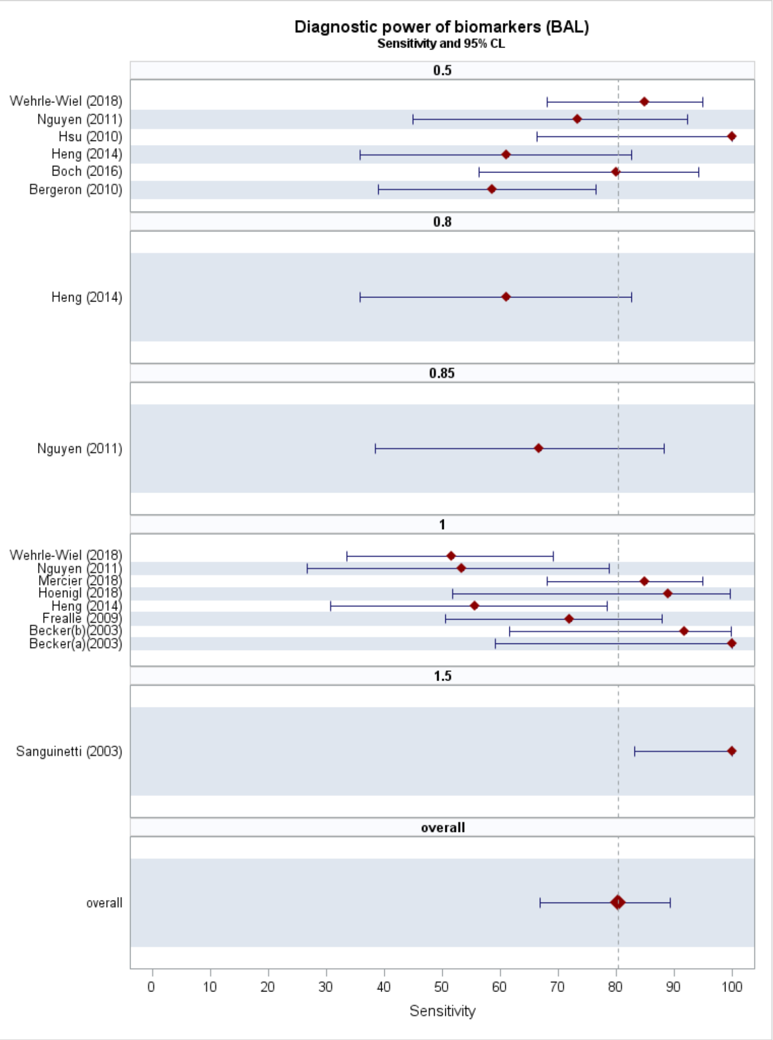

Figure S15 – Forest plot of diagnostic power of specificity BAL galactomannan as a diagnostic assay for proven/probable IPA versus no-IPA.

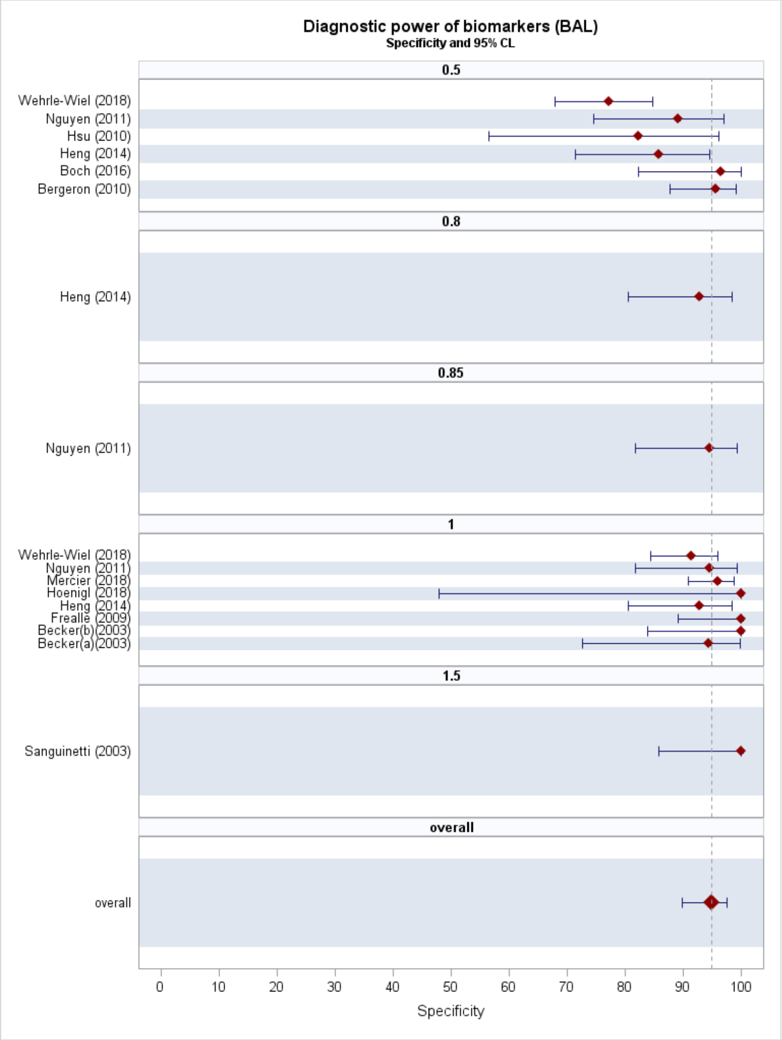

Figure S16 – Forest plot of diagnostic power of sensitivity BAL galactomannan as a diagnostic assay for proven/probable/possible IPA versus no-IPA.

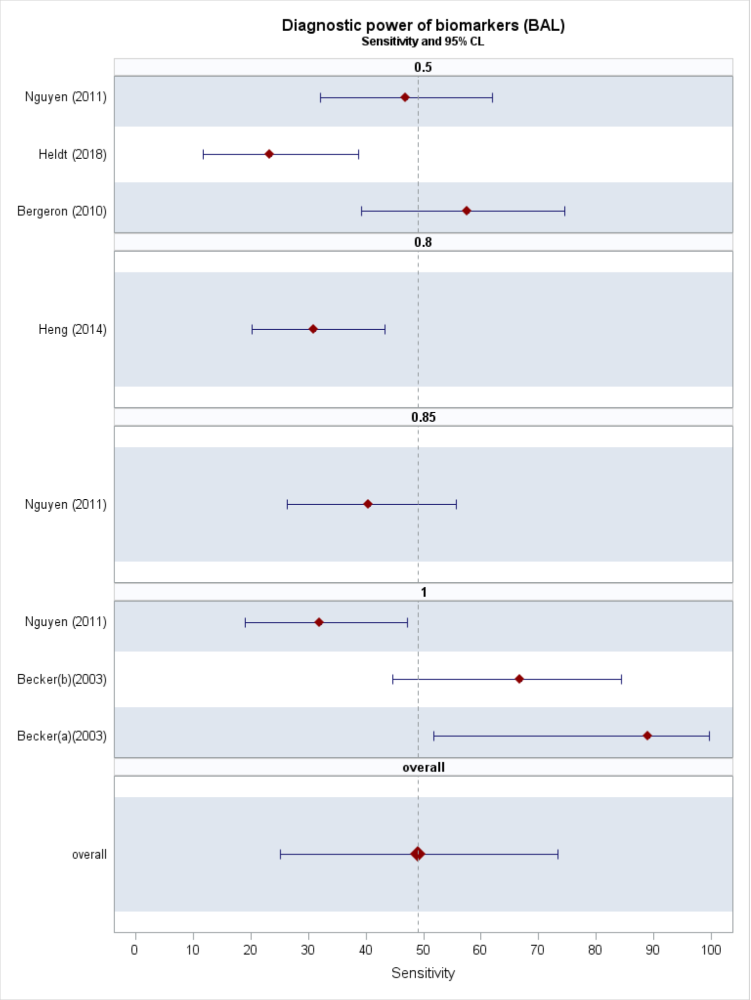

Figure S17 – Forest plot of diagnostic power of specificity BAL galactomannan as a diagnostic assay for proven/probable/possible IPA versus no-IPA.

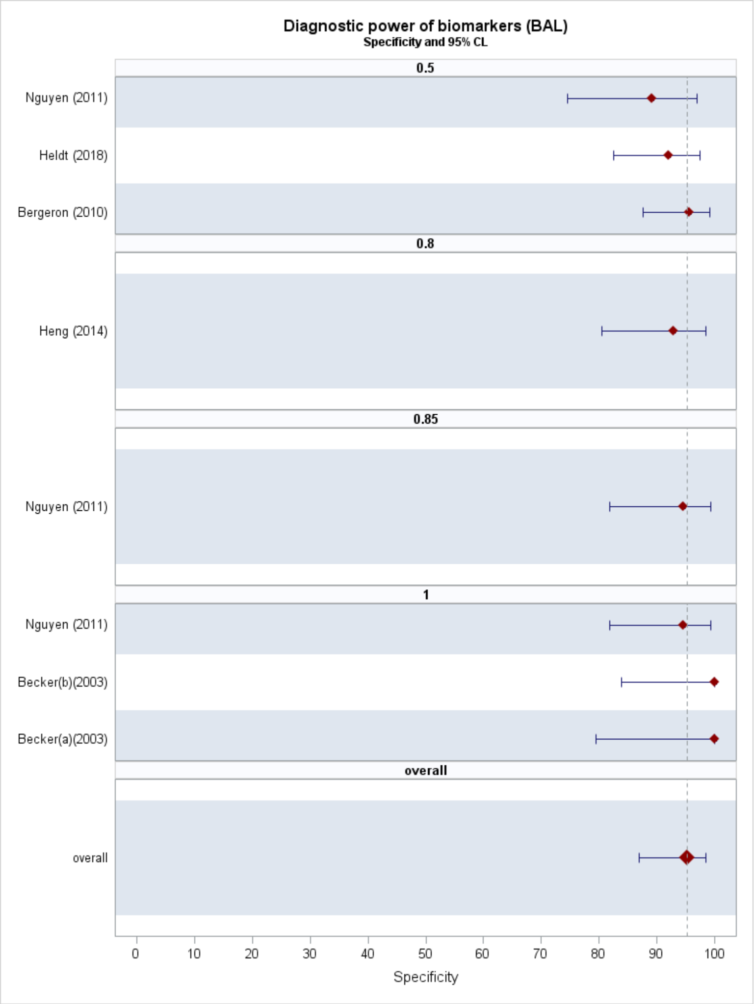

Figure S18 – Forest plot of diagnostic power of sensitivity BAL galactomannan as a diagnostic assay per cut-off proven/probable IPA versus no-IPA.

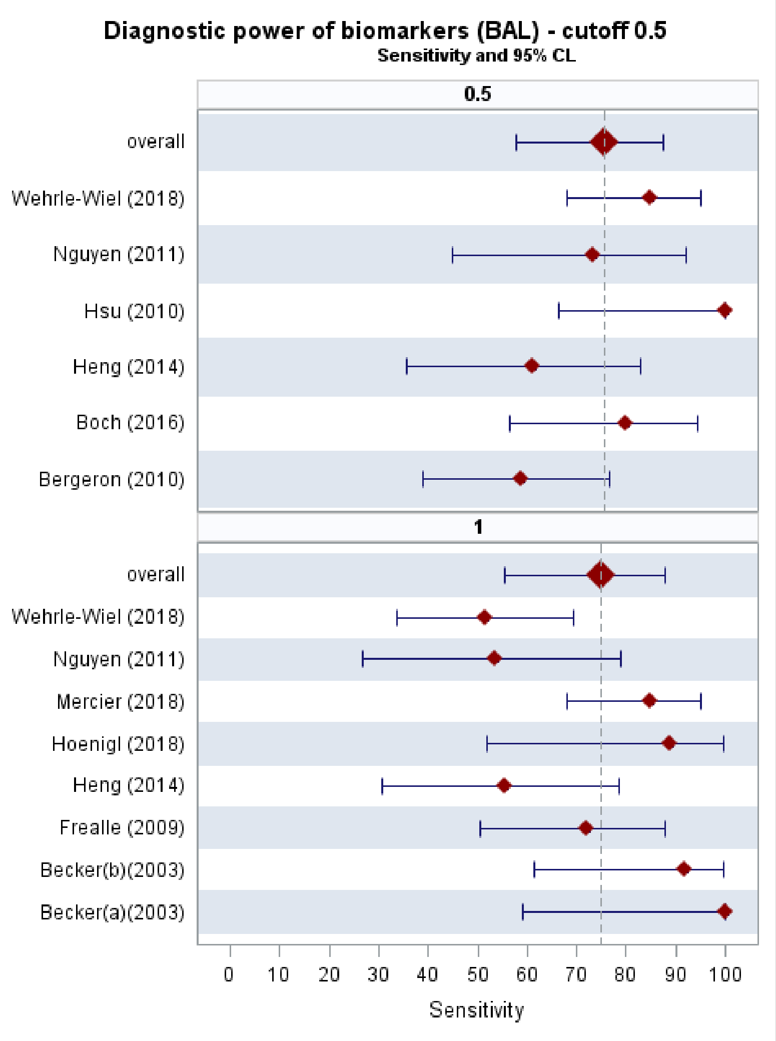

Figure S19 – Forest plot of diagnostic power of sensitivity BAL galactomannan as a diagnostic assay per cut-off proven/probable IPA versus no-IPA.

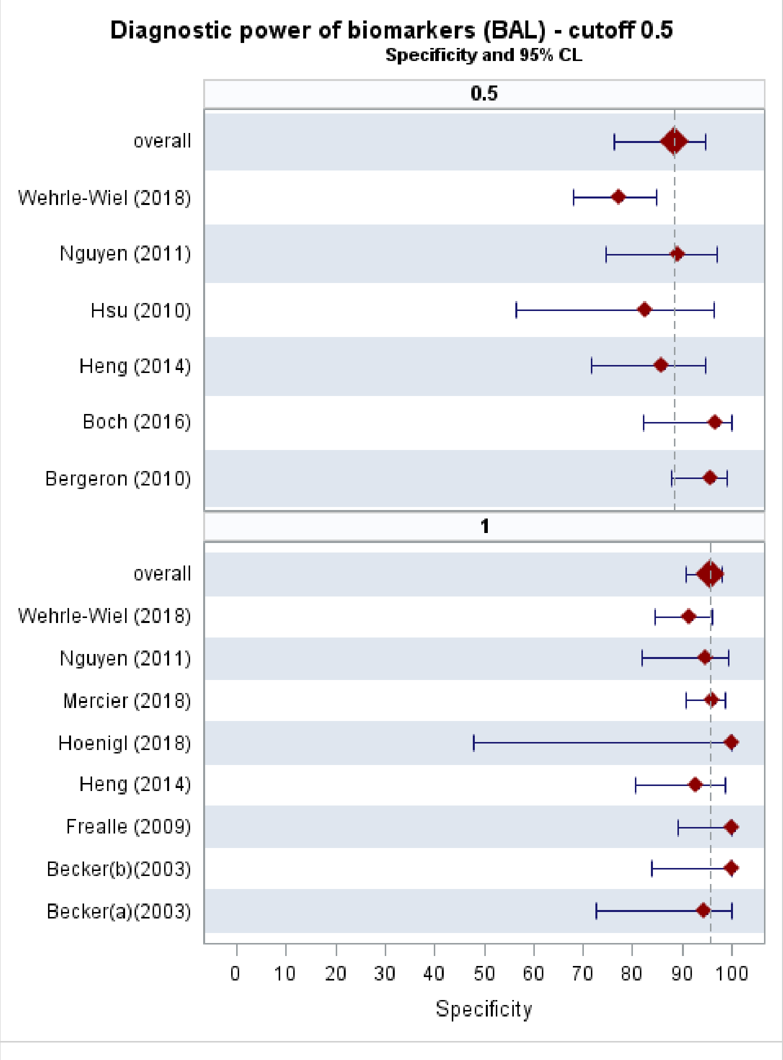

Table S15 - Pooled results of the included studies for BAL GM for proven/probable IPA vs. no IPA

Cut-off value	Nr. of studies	Sensitivity [95% CI]	Specificity [95% CI]
**0.5**	6	0.75 [0.58-0.87]	0.88 [0.76-0.95]
**0.8**	1	0.61 [0.36-0.83]	0.93 [0.81-0.99]
**0.85**	1	0.67 [0.39-0.88]	0.95 [0.82-0.99]
**1.0**	8	0.75 [0.56-0.88]	0.96 [0.91-0.98]
**1.5**	1	100.00 [0.83-100.00]	100.00 [0.86-100.00]

Test difference in using
Sensitivity: pvalue=0.5347
Specificity: pvalue=0.0863

Figure S20 – Forest plot of diagnostic power of sensitivity BAL galactomannan as a diagnostic assay per cut-off proven/probable/possible IPA versus no-IPA.

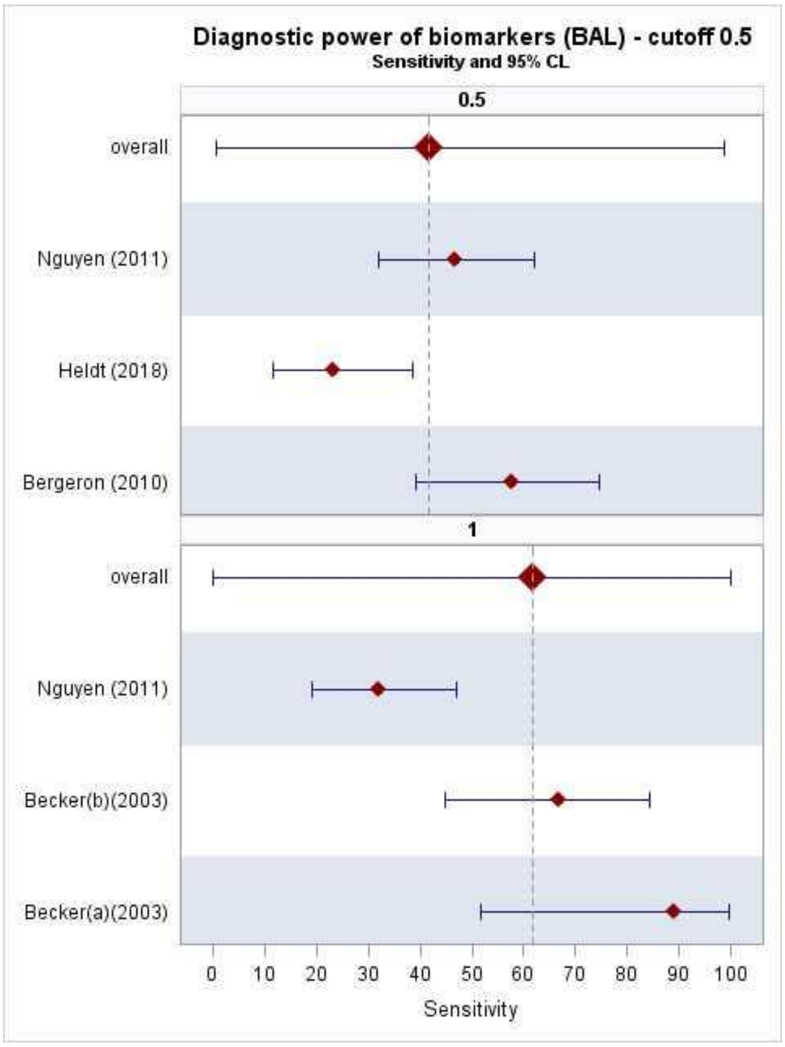

Figure S21 – Forest plot of diagnostic power of specificity BAL galactomannan as a diagnostic assay per cut-off proven/probable/possible IPA versus no-IPA.

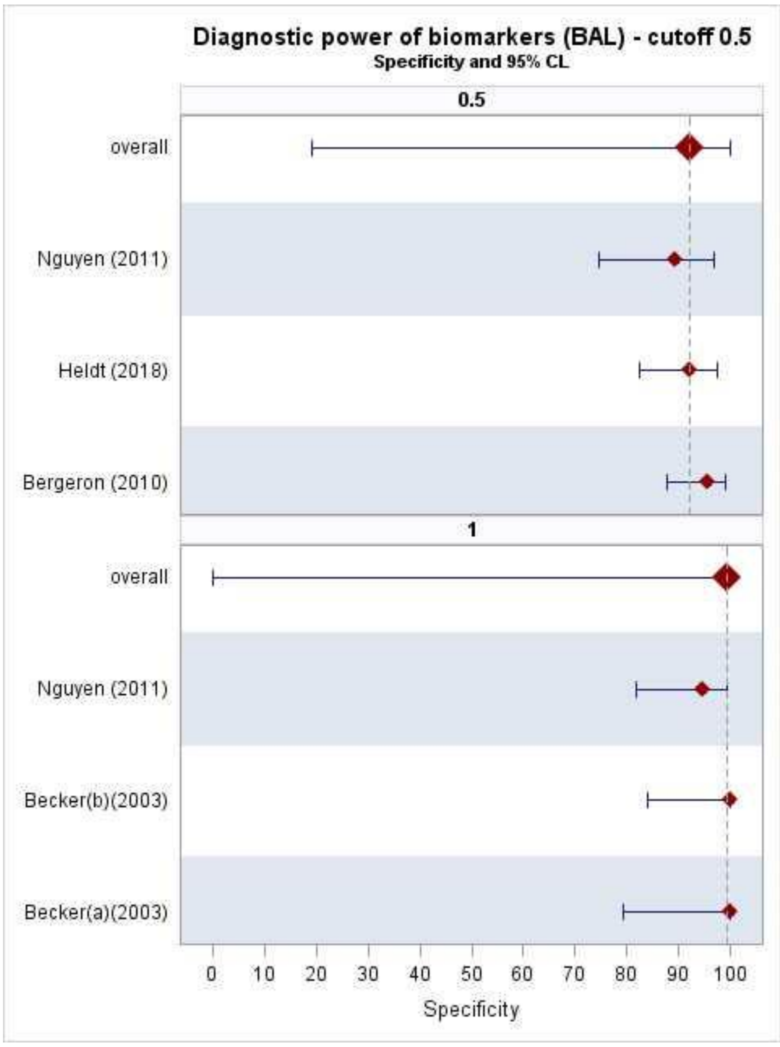

Table S16 - Pooled results of the included studies for BAL GM for proven/probable/possible IPA vs. no IPA

**Cut-off value**	**Nr. of studies**	**Sensitivity [95% CI]**	**Specificity [95% CI]**
**0.5**	3	0.42 [0.73-0.99]	0.92 [0.19-0.99]
**0.8**	1	0.31 [0.20-0.43]	0.93 [0.81-0.99]
**0.85**	1	0.40 [0.26-0.56]	0.95 [0.82-0.99]
**1.0**	3	0.62 [0.03-1.0]	0.99 [0.00-1.0]
